# Role of Vision and Mechanoreception in Bed Bug, *Cimex lectularius* L. Behavior

**DOI:** 10.1371/journal.pone.0118855

**Published:** 2015-03-06

**Authors:** Narinderpal Singh, Changlu Wang, Richard Cooper

**Affiliations:** Department of Entomology, Rutgers University, New Brunswick, New Jersey, United States of America; University of Cincinnati, UNITED STATES

## Abstract

The role of olfactory cues such as carbon dioxide, pheromones, and kairomones in bed bug, *Cimex lectularius* L. behavior has been demonstrated. However, the role of vision and mechanoreception in bed bug behavior is poorly understood. We investigated bed bug vision by determining their responses to different colors, vertical objects, and their ability to detect colors and vertical objects under low and complete dark conditions. Results show black and red paper harborages are preferred compared to yellow, green, blue, and white harborages. A bed bug trapping device with a black or red exterior surface was significantly more attractive to bed bugs than that with a white exterior surface. Bed bugs exhibited strong orientation behavior toward vertical objects. The height (15 vs. 30 cm tall) and color (brown vs. black) of the vertical object had no significant effect on orientation behavior of bed bugs. Bed bugs could differentiate color and detect vertical objects at very low background light conditions, but not in complete darkness. Bed bug preference to different substrate textures (mechanoreception) was also explored. Bed bugs preferred dyed tape compared to painted tape, textured painted plastic, and felt. These results revealed that substrate color, presence of vertical objects, and substrate texture affect host-seeking and harborage-searching behavior of bed bugs. Bed bugs may use a combination of vision, mechanoreception, and chemoreception to locate hosts and seek harborages.

## Introduction

Behaviors during host finding in haematophagous insects are typically dominated by responses to visual, thermal, and olfactory cues [1], [2]. These multimodal sensory cues improve their chances of host finding [2]. Attraction to thermal [3–5] or olfactory cues such as carbon dioxide (CO_2_), kairomones [3–8], and pheromones [9–12] has been demonstrated in bed bugs, *Cimex lectularius* L. (Heteroptera: Cimicidae). However, the role of other sensory modalities such as vision and mechanoreception in bed bugs has not been well documented.

Most insects have three types of spectral photoreceptors with peak sensitivities in the ultraviolet (350 nm), blue (440 nm), and green (530 nm) part of the spectrum [13]; however, photoreceptors sensitive to red (600 nm) have been reported in the Odonata, the Hymenoptera, the Lepidoptera, and the Coleoptera [14]. These chromatic signals are used by insects for several behaviors such as oviposition, host searching, pollination, object detection, pattern recognition, and motion vision [15–17]. Exploitation of visual cues in insect monitoring fall into three categories: light that attracts insects, colored objects that are attractive due to their specific reflectance, and shapes that stand out against a contrasting background [18], [19]. The preference for trap color, shape [18], [20–24], and texture of the outer surface [25–27] has been exploited to monitor and manage many economically and medically important insect pests.

Color preference in insects is well documented [14]. Red light was more attractive to the kissing bug, *Triatoma infestans* (Klug) followed by blue and then by green in the presence of chemical attractants [28], [29]. Koehler et al. [30] showed the effect of different spectral lights (ultraviolet, green, gold or red) on locomotor activity of the German cockroach, *Blattella germanica* L. Black, blue, and red colors were attractive to black flies (*Simulium* and *Prosimulium* spp.) [31] and tsetse flies (*Glossina* spp.) [32–34]. Red and blue colored plasticized corrugated boards were more attractive to adult stable flies, *Stomoxys calcitrans* L. than orange and white boards [35]. The larvae and adults of the red flour beetle, *Tribolium castaneum* (Herbst) significantly preferred colored surfaces over white surfaces [36]. Color based visual cues may also play an important role in the host searching behavior of bed bugs; however, research on color preference with this insect is lacking.

Attraction to vertical objects that mimic a host has been demonstrated in Warren root collar weevil, *Hylobius warreni* (Wood) [37] and the melon fly, *Bactrocera cucurbitae* (Coquillett) [38]. Adults of the red flour beetle were more likely to visit black pillars against a white background than white pillars, and the number of visits to black pillars increased with pillar height [18]. Multiple-funnel traps provided an attractive vertical silhouette, similar to a host stem, for capturing the southern pine beetle, *Dendroctonus frontalis* (Zimmermann) [22]. Bed bugs climb onto furniture legs in order to reach a sleeping human host for blood meals. Whether bed bugs use vertical objects as visual cues for finding their host is unknown.

Insects also have background light thresholds necessary to perceive and discriminate different colors [39], [40]. Higher numbers of red flour beetles were captured in traps in front of black panels under both high (2160 lx) and low light intensity (98 lx) conditions, but not under dark (0 lx) conditions [18]. There is no information available on the ability of bed bugs to perceive colors and objects under different light conditions.

Since bed bugs are thigmotactic [11], [41], the tactile stimulus generated from antennal, tarsal, and body sensory hairs [42–46] contacting the outer texture of any object can cause a change in their behavior or movements. Bed bugs use their hook-like tarsal claws to grip and climb vertical surfaces [47]. It is shown that bed bugs have a distinct preference for rough surfaces compared to smooth surfaces (brick floor vs. lime or sandy floor material, filter paper vs. glazed paper, woolen stockinet vs. cotton stockinet or silk, and rough wood vs. smooth wood) [44]. Findings from these studies suggest that the substrate texture can significantly affect orientation behavior of bed bugs. Many bed bug monitoring devices have been developed in recent years [48]; however, their efficacies vary significantly [49]. Understanding how subtle differences in texture of a trap affect its efficacy is an interesting subject of research that will help design more effective bed bug monitors.

In this study, we determined the effect of vision (color, vertical object, and light intensity) and mechanoreception (texture) on bed bug orientation behavior. The findings provide important scientific basis for designing more effective bed bug monitoring devices.

## Materials and Methods

### Insects

Bed bugs were collected from infested apartments in Newark, NJ 2–3 months prior to this study. Permission was obtained from the housing management for collecting bed bugs. They were maintained in plastic containers (4.7 cm height and 5 cm diameter) with folded paper as harborages at 26 ± 1°C, 40 ± 10% relative humidity (RH), and a 12:12 h (L:D) photoperiod. Bed bugs were fed weekly on defibrinated rabbit blood (Hemostat Laboratories, Dixon, CA) using a Hemotek membrane-feeding system (Discovery Workshops, Accrington, UK). Since the blood was purchased from a commercial source, ethics approval from the Institutional Review Board was not necessary. Bed bugs were starved for one week prior to bioassays [50]. Starved bed bugs are less likely to aggregate than the recently fed bed bugs [51]. Only males and 3^rd^ to 5^th^ instar bed bug nymphs were used in this study. All the nymphs molted during the starvation week before being used in the experiments. Females were not tested to avoid egg-laying in the arenas.

### Test arenas and experimental conditions

#### a. Plastic dishes

**Fig 1:**
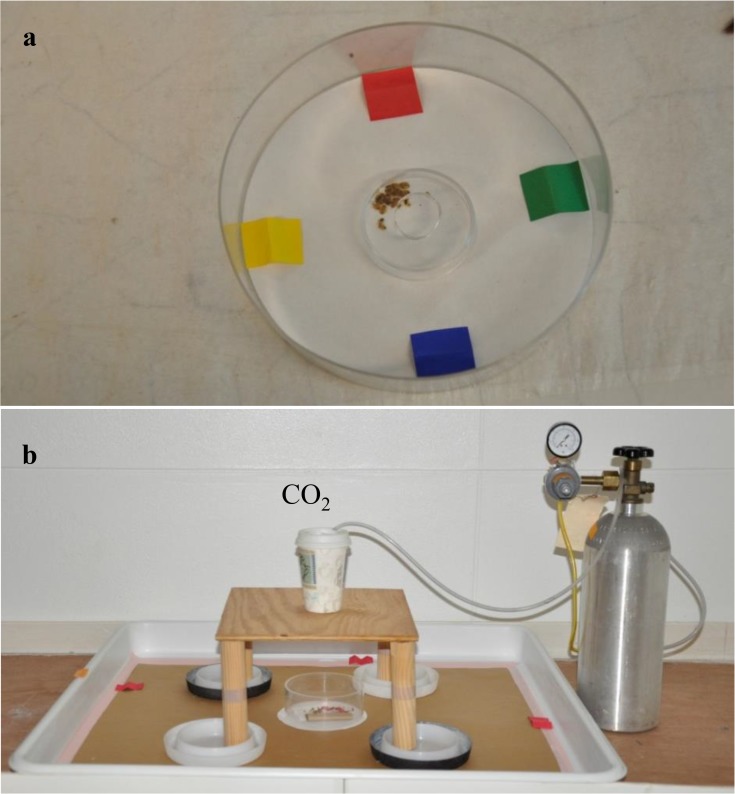
Experimental setup for determining bed bug preference to colors. a) colored harborages, b) colored interceptors.

Plastic dishes (11.5 cm diameter and 3.75 cm height) with bottoms lined with 11.0 cm diameter filter paper (VWR International, Arlington Heights, IL) were used. This experiment was conducted in a 4 m long and 2.3 m wide ventilated room with white walls (background color) under low background light (0.05–0.5 lx), 26 ± 1°C temperature, and 40 ± 10% RH. Bed bugs were confined at the center of each dish for 30 min with a plastic ring (3.5 cm diameter and 1 cm height). The ring was then removed allowing bed bugs to move to any harborage (Fig. 1A). The bed bug locations and numbers were recorded after 4 h with the aid of a red light [11]. Twenty 3^rd^ to 5^th^ instar bed bug nymphs were used in each replication (dish).

#### b. Plastic white tray arenas

White ClimbUp Insect Interceptors (Susan McKnight, Inc., Memphis, TN), referred to hereafter as white interceptors (15.24 cm diameter and 2.54 cm height) were used. The interior surface of the interceptors was coated with a light layer of fluoropolymer resin (BioQuip products, Rancho Dominguez, CA) to prevent trapped bed bugs from escaping. White plastic tray arenas (80 by 75 by 5 cm) (L × W × H) with bottoms lined with brown paper were used (Fig. 1B). A layer of fluoropolymer resin was applied to inner walls of the arenas to prevent the bed bugs from escaping. A filter paper (15 cm diameter) was placed at the center of each arena and then a plastic ring (13.3 cm diameter and 6.4 cm height) was placed on the filter paper for confining the bed bugs. A piece of folded cardboard and folded fabric was placed on the filter paper to provide harborages for bed bugs. Four additional red colored paper harborages (5.1 cm long and 3.3 cm wide) were placed along the edges on the floor of each tray arena. These harborages were exposed to bed bugs for 3–4 weeks prior to the bioassays and contained bed bug feces and exuvia similar to the natural harborages of bed bugs in their habitat. Additional harborages provided more hiding or resting places for bed bugs. Fifty bed bugs (25 3^rd^ to 5^th^ instar bed bug nymphs, 25 adult males) were released into each arena and confined with a plastic ring. The bed bugs were acclimated for approximately 15 h before removing the plastic ring. At 1 h into the dark cycle, CO_2_ (100 ml/min) was introduced from a 5 lb cylinder (Airgas East Inc., Piscataway, NJ) into 240 ml plastic cups that were placed on the top of the stool (only in color preference or texture preference experiments) or in the center of room on a 1.2 m tall stool. CO_2_ was used to stimulate the bed bug foraging activity [8], [52]. The plastic ring confining the bed bugs was removed. The numbers of bed bugs trapped in the interceptors and those in the arenas were collected and counted after 8 h with the aid of a red light. After counting, dead and moribund bed bugs were taken out and at least 50% of the bed bugs were replaced with new bed bugs in each arena. Bed bugs that showed no visible signs of decreased activity were re-used due to limited number of bed bugs with similar feeding status and physiological stage. All bed bugs were placed back to the center of the arenas and confined with plastic rings for 15 h before starting the next bioassay.

### Color preference

#### a. Colored harborages

Folded paper harborages (2.0 cm long × 1.5 cm wide) were made from colored construction papers (Universal Stationers Supply Co., Deerfield, IL). Two groups each containing four different color harborages were tested separately: 1) black, white, green, and yellow, and 2) red, blue, green, and yellow. Order of the color harborages was randomized within each dish. Paper harborages were placed on edges in the dish equidistant from each other (Fig. 1A). Each group was replicated four times.

Black and red colors were the most preferred colors from the above experiment. Therefore, another bioassay was conducted to determine if bed bugs show a preference to black or red color. In each plastic dish, either black or red and a white harborage (control), was placed along the edge of the dish opposite to each other. Each color was replicated four times.

#### b. Colored intercepting devices

This experiment was conducted to further investigate if bed bugs have a preference to black or red color and also to determine if bed bug behavioral preference to red or black exterior surface can be used to enhance trap efficacy. The exterior of white interceptors were painted black and red with spray paint (ColorPlace, Walmart, Bentonville, AR). A wooden stool (26.5 cm long × 26.5 cm wide) with four legs was placed in center of each arena (Fig. 1B). Prior to releasing the bed bugs, two colored (either black or red) and two white interceptors (control) were placed under four stool legs with the same color diagonally opposite to each other (Fig. 1B). Six arenas were used and on each day, three arenas were used for each color. The locations of the red and black colored interceptors in each arena were switched the next day. Thus, each color was replicated six times over two consecutive days.

### Response to vertical objects of various shapes, colors, and heights

**Fig 2:**
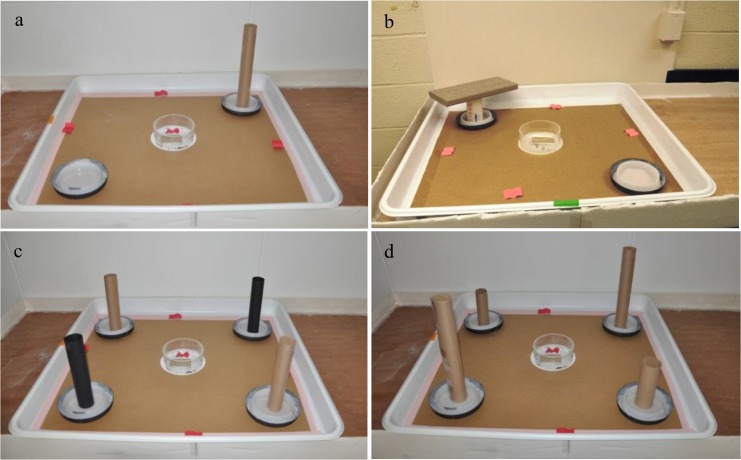
Experimental setup for determining the response of bed bugs to vertical objects. a) tubular object, b) rectangular object, c) brown tubular object vs. black tubular object, and d) 30 cm vs. 15 cm tall brown tubular object.

Four experiments were conducted to determine whether bed bugs orient toward vertical objects. Fiebing's black leather dye (Tandy Leather Factory, Fort Worth, TX) was applied to the exterior surface of white interceptors to produce black interceptors. Black interceptors were used in white plastic tray arenas (Fig. 2). Both brown and black vertical tubular objects (30 cm tall, 5 cm diameter) were made from construction paper. The brown tubular objects were included because brown color represents the typical color of furniture legs in residential settings. The vertical objects tested were:

#### a. Presence of a tubular vertical object

Three arenas were used and in each arena two interceptors were placed diagonally at two corners equidistant (25 cm) from the center. One interceptor contained a vertical brown tubular object and the other interceptor (control) did not contain any object (Fig. 2A). This experiment was repeated the next day yielding six replicates. The positions of the two interceptors in each arena were switched after 1 d.

#### b. Presence of a rectangular vertical object

A similar setup as the previous experiment was used to evaluate the effect of a rectangular object (26 × 18 × 3 cm) mounted on a 15 cm tall and 5.5 cm diameter white plastic pipe (Fig. 2B). This rectangular object simulates the horizontal orientation of a bed atop of bed frame legs. One interceptor contained a rectangular object and the other interceptor (control) did not contain a rectangular object. This experiment was replicated six times during two consecutive days. The positions of two interceptors in each arena were switched after 1 d.

#### c. Object color

Black and brown tubular objects (30 cm tall, 5 cm diameter) were simultaneously evaluated in four tray arenas. In each arena, four interceptors were placed diagonally at four corners equidistant (25 cm) from the center. Two diagonally placed interceptors received brown tubular objects and the other two interceptors received black tubular objects (Fig. 2C). This experiment was repeated the next day yielding eight replications. The positions of brown and black objects in each arena were switched after 1 d.

#### d. Object height

No significant difference between the brown or black object was observed in previous experiment. So, the brown tubular object was selected to determine the effect of object height. Using the setup described in the previous experiment, 30 cm tall objects (5 cm diameter) were placed in two diagonally placed interceptors and 15 cm tall objects (5 cm diameter) were placed in the other two interceptors (Fig. 2D). This experiment was repeated the next day yielding eight replications. The positions of the 30 and 15 cm objects in each arena were switched after 1 d.

### Effect of light intensity

Two background light intensities were tested: complete darkness (0 lx) and low light intensity (0.05 lx). The low light intensity in the room was maintained by covering a 13 W fluorescent lamp (Model # 230128; Philips; Andover, MA) with three layers of 100% white cotton cloth. The light source was placed in the center of the room so that all arenas receive similar light intensity. The background light intensity was measured using an EA33 EasyView light meter (Extech Instruments Corporation, Nashua, NH). Four white plastic tray arenas were used. To examine the effect of light intensity on color preference, one black interceptor and one white interceptor were placed diagonally at two corners of plastic tray arena equidistant (25 cm) from the center. To examine the effect of light intensity on bed bug orientation behavior toward a vertical object, two black interceptors were placed in each arena. One interceptor received a 30 cm tall brown tubular object and the other interceptor (control) did not receive any object (Fig. 2A). In both tests, each light intensity was replicated eight times during two consecutive days. The positions of two interceptors in each arena were switched after 1 d.

### Texture preference

Black texture paint (Rust Oleum, Vernon Hills, IL) and black polyester felt were used on white interceptors with the paper surgical tape removed to create two new textures. Black spray paint and Fiebing's black leather dye were applied to the white interceptors with the white paper surgical tape on to produce another two textures. They were compared with the white interceptors which had a white paper surgical tape covering the exterior walls. These textures provided a diverse range varying from smooth to rough surfaces to determine bed bug responses to different textures. The setup was the same as Fig. 1B but with a different exterior texture on each interceptor. These five textures were tested in two combinations: 1) white paper surgical tape (white tape), white paper surgical tape dyed black (dyed tape), white paper surgical tape painted black (painted tape), black polyester felt (felt); 2) dyed tape, painted tape, felt, black texture paint on plastic (textured painted plastic). Four plastic arenas were used each day for testing each group. Each group was replicated eight times during four consecutive days of testing.

### Statistical analyses

Bed bug distribution among interceptors (experiments in white plastic trays) or harborages (experiments in plastic dishes) in each experimental arena was summarized as percentage of bed bugs in interceptors or harborages and percentage of bed bugs remained in the arena. The mortality was low (< 5%) in all experiments. Therefore, the interceptor or harborage counts were not adjusted for the mortality. These experiments were not designed to compare responses between nymphs and adults; therefore, the combined counts (adults and nymphs) were used for data analysis. Bed bug counts in interceptors or harborages were analyzed using PROC GLIMMIX in SAS version 9.3 (SAS Institute, Cary, NC). The model accommodates random effects, repeated measures, and overdispersion. Plastic dish or arena was included as random effect. Depending on the experiment the fixed effect in the model was color, vertical object or texture. Binomial distribution with logit or multinomial distribution with glogit link function along with maximum pseudo-likelihood estimation was used. Contrast statements were used to perform multiple comparisons. In all experiments, only those bed bugs that appeared in the interceptors or harborages were analyzed.

## Results

### Color preference

**Fig 3:**
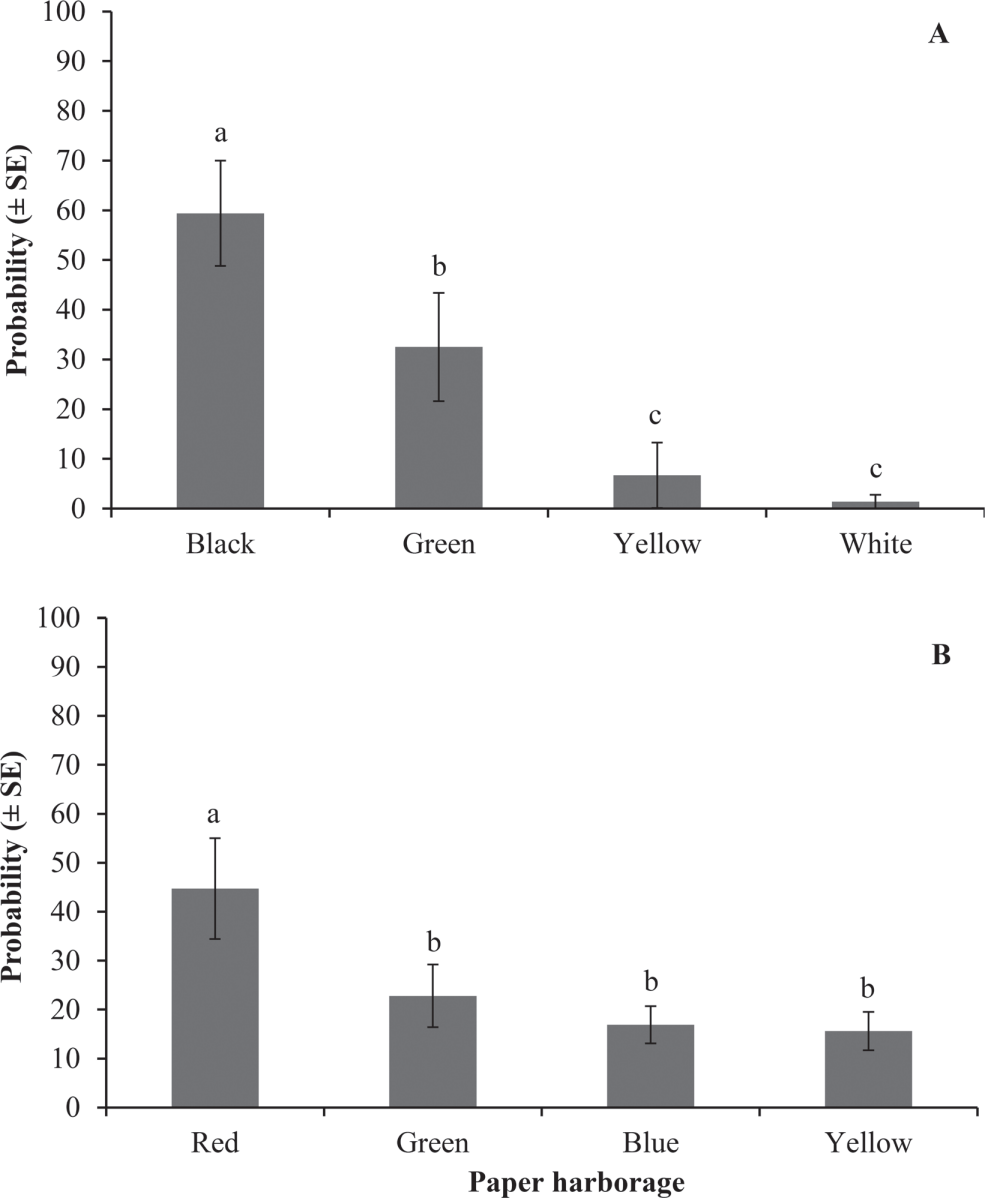
Probability of bed bugs staying under colored paper harborages. A) black, green, yellow, and white, and B) red, green, blue, and yellow. Twenty bed bug nymphs were used in each replication and each group was replicated four times. Different letters above the bars indicate significant differences (*P* < 0.05).

The color of the paper harborage had a significant effect on bed bug behavior. There were significant differences in bed bug numbers staying under black, green, yellow, and white paper harborages (F = 6.3; df = 3, 12; *P* = 0.0003). In each test arena, the probability (mean ± SE) of bed bugs staying under black, green, yellow, and white paper harborages was 59.4 ± 10.6, 32.5 ± 10.9, 6.7 ± 6.6, and 1.4 ± 1.4%, respectively. Black color was significantly more attractive than other colors tested. Green was more attractive than yellow and white; however, yellow and white showed the same probability of harboring bed bugs (Fig. 3A). In the second color group study, the probability of bed bugs staying under red, green, blue, and yellow paper harborages was 44.7 ± 10.3, 22.8 ± 6.4, 16.9 ± 3.8, and 15.6 ± 3.9%, respectively. Red color was significantly more attractive than other colors (F = 7.3; df = 3, 12; *P* = 0.0001) (Fig. 3B).

When black or red color harborages were compared against white harborages, the probability of bed bugs staying under black (85.2 ± 4.8%) was significantly (F = 4.6; df = 1, 6; *P* = 0.0001) higher than that staying under white color (14.8 ± 4.8%). Similarly red (77.4 ± 5.7%) was significantly (F = 3.8; df = 1, 6; *P* = 0.0002) more attractive than white color (22.6 ± 5.7%). The probability of trapping bed bugs in black interceptors (68.0 ± 3.1) was significantly (F = 5.3; df = 1, 10; *P* = 0.0001) higher than that trapped in white interceptors (32.0 ± 3.1). Similarly significant differences (F = 1.2; df = 1, 10; *P* = 0.04) were found in the probability of trapping bed bugs between red (54.5 ± 4.0%) and white interceptors (45.5 ± 4.0%).

### Response to vertical objects of various shapes, colors, and heights

The presence of a vertical object influenced bed bug orientation behavior. A significant difference was found in the probability of trapping bed bugs between the interceptor with a brown tubular object (62.5 ± 3.1%) and the control interceptor (37.5 ± 3.1%) (F = 3.8; df = 1, 10; *P* = 0.0001). Similarly, significantly more bed bugs went into the interceptor containing a rectangular object (64.4 ± 2.9%) than those in the control interceptor (35.6 ± 2.9%) (F = 4.6; df = 1, 10; *P* = 0.0001). No significant differences were found in the probability of bed bugs being trapped in the interceptors containing black (41.2 ± 2.2%) or brown tubular objects (48.8 ± 2.2%) (F = 0.8; df = 1, 14; *P* = 0.41). The probability of bed bugs being trapped in the interceptor containing 15 cm (50.9 ± 2.2%) or 30 cm tall brown tubular object (49.1 ± 2.2%) was not significantly different (F = 0.4; df = 1, 14, *P* = 0.68).

### Effect of light intensity

The probability of trapping bed bugs in the black (53.9 ± 2.8%) and white interceptors (46.1 ± 2.8%) was not significantly different under complete dark conditions (F = 1.4; df = 1, 14; *P* = 0.17). Whereas, the probability of trapping bed bugs in the black (70.0 ± 2.5%) and white interceptors (30.0 ± 2.5%) was significantly different under low light conditions (F = 7.0; df = 1, 14; *P* = 0.0001). Overall trap catch rate (bed bugs in black interceptor plus white interceptor divided by total bed bugs present in each arena) under complete dark and low light conditions was 78% and 81%, respectively.

Similar to the color test, under complete dark conditions, no significant difference (F = 1.3; df = 1, 14; *P* = 0.19) was found in the probability of trapping bed bugs in the interceptor containing the brown tubular object (42.9 ± 5.0%) and control interceptor (57.1 ± 5.0%). Whereas, the brown tubular object (82.4 ± 2.9%) and control interceptors (17.6 ± 2.9%) were significantly different in their probability of trapping bed bugs under low light conditions (F = 7.6; df = 1, 14; *P* = 0.0001). Overall trap catch rate (brown tubular object plus control interceptor divided by total bed bugs present in each arena) under complete dark and low light conditions was 36% and 49%, respectively.

### Texture preference

**Fig 4:**
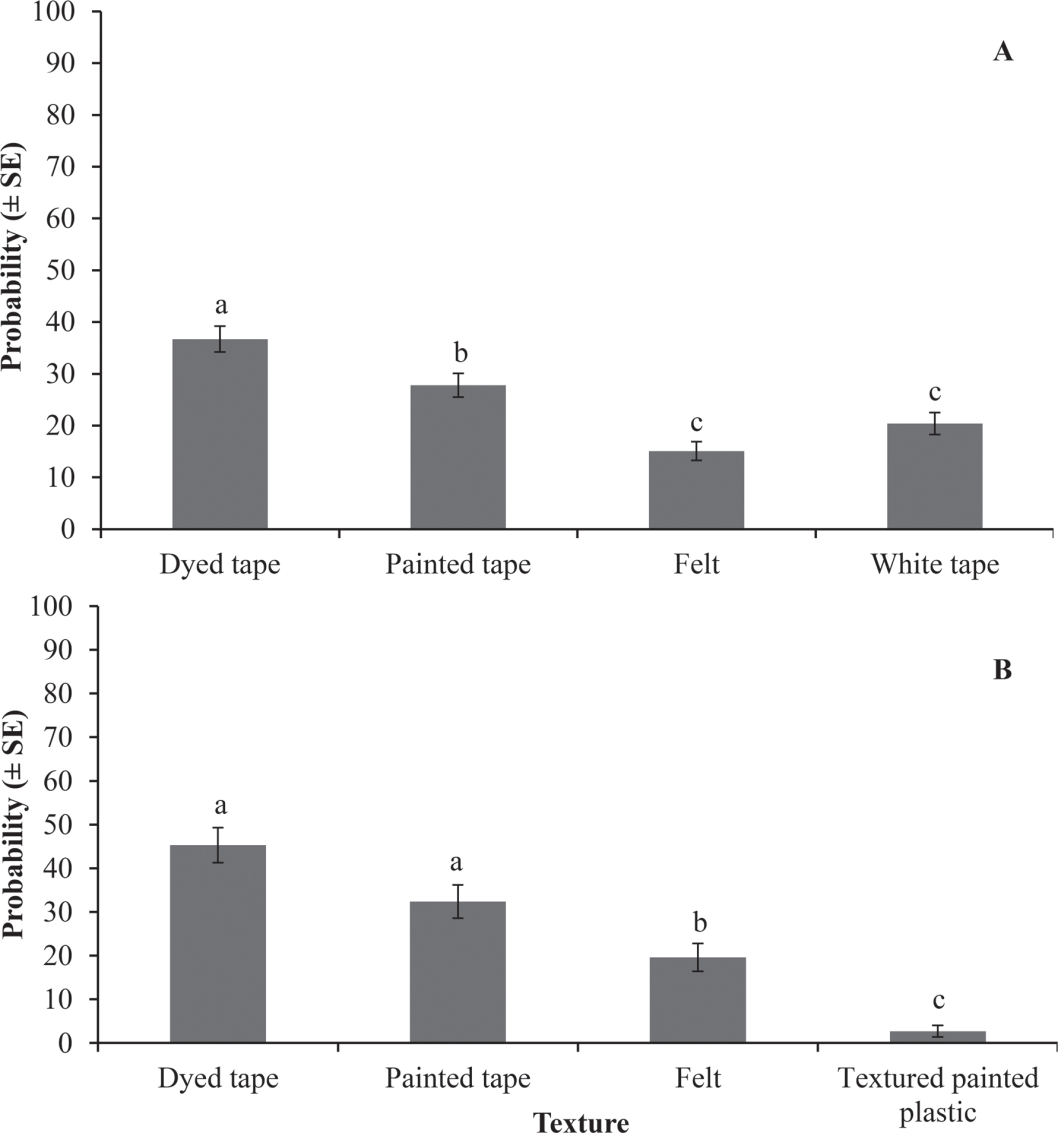
Probability of trapping bed bugs in interceptors with different exterior textures. A) white paper surgical tape dyed black (dyed tape), white paper surgical tape painted black (painted tape), black polyester felt (felt), and white paper surgical tape (white tape), and B) dyed tape, painted tape, felt, and black texture paint on plastic (textured painted plastic). Fifty bed bugs (25 nymphs and 25 adult males) were used in each replication and each group was replicated eight times during four consecutive days of testing. Different letters above the bars indicate significant differences (*P* < 0.05).

In the first combination test, bed bugs showed a significant preference among different textures tested (F = 12.5; df = 3, 28; *P* = 0.0001). The probability of bed bugs being trapped in a dyed tape, painted tape, felt, and white tape interceptors was 36.7 ± 2.5, 27.8 ± 2.3, 15.1 ± 1.8, and 20.4 ± 2.1%, respectively. Dyed tape was the most preferred texture (Fig. 4A). In the second combination test, dyed tape, painted tape, felt, and textured painted plastic interceptors were significantly different in their probability of trapping bed bugs: 45.3 ± 4.0, 32.4 ± 3.8, 19.6 ± 3.2, and 2.7 ± 1.3%, respectively (F = 13.1; df = 3, 28; *P* = 0.0001). Dyed tape and painted tape were statistically more favored than felt and textured painted plastic (Fig. 4B).

## Discussion

Color vision is the ability of an animal to distinguish two monochromatic spectral lights or colors on the basis of wavelength differences regardless of their relative intensity [15], [53]. In insects, color vision tends to move towards ultraviolet, and most are generally considered essentially blind to red color [13]. However, red color perception has been reported in some insects [14] including the nocturnal kissing bug [28], [29] which has a similar natural history and behavior as bed bugs. So far, nothing is known on spectral sensitivity of photoreceptors in bed bugs. Our studies revealed some interesting aspects of bed bug orientation behavior. Bed bug nymphs demonstrated distinct color preferences orienting to black and red colors the most. Whether bed bugs can differentiate red or black color is not known. It is likely that the bed bugs may perceive red and black colors as dark colors only and thus prefer to settle down under dark colors due their photonegative reaction. A strong preference to black color has also been shown in the kissing bug [28], [29]. Black color is recognized as the preferred color by a number of other insects. Reza and Parween [36] showed red flour beetle larvae tend to aggregate only on black surfaces. Red flour beetle adults were more likely to visit black pillars than white pillars when presented with a choice [18]. Kusakabe and Ikeshoji [54] found *Aedes aegypti* mosquitoes to be more attractive to black color under laboratory conditions. *Aedes* spp. and *Mansonia perturbans* preferred black, red, and blue over white and yellow in cuboid funnel traps [55]. To answer whether bed bugs can discriminate between different colors, the photoreceptors sensitivity to different colors of varying wavelengths and intensities needs to be established.

The preference for red and black color also needs to be considered from bed bug relative perception to these dark colors through color contrast phenomenon. These experiments were conducted against a white background that made the dark colored (red, black) objects (harborages or interceptors) stand out more strongly than the light colored objects (white, yellow). The contrast between the dark colors and the white background may have accounted for the bed bug preference to the dark colors. This visual color contrast has been shown in honey bees, *Apis mellifera* L., where blue color on a yellow background looked bluer to the bee than a blue color on a grey background. Likewise a yellow color on a blue appeared more yellow than if it were on a grey background [13]. Color contrast has been shown to aid honey bees, ants, and many other pollinators to navigate to landmarks [56], [57] and to effectively find colored flowers against the background of green foliage [58], [59]. Multicolored Asian lady beetle, *Harmonia axyridis* (Pallas) use visual contrast in their choice for overwintering sites [60]. The red flour beetle adults preferred black shapes to white shapes against a white background [18]. Majority of the apartment buildings usually have light colored walls and floors. The preference for black color in bed bugs suggests incorporating black color in designing bed bug monitors may help improve trap efficacy but further field testing is warranted. Whether bed bugs use color contrast phenomenon for detecting colors, objects or even hosts still needs to be explored further using different combinations of colors and backgrounds.

Another interesting finding of this study is that bed bugs orient to vertical objects which may play a role in guiding their foraging behavior. However, the color and height of vertical objects did not have any significant effect on trap catch, suggesting the tendency of bed bugs to move up to any vertical object tested in this study. Significant effect on trap catch in presence of vertical brown or black objects against the light background further suggests the possibility of using visual color contrast in discriminating objects by bed bugs. Our findings have practical implications in bed bug management. Placing a vertical object in a bed bug monitor will likely increase the efficacy of the monitor. Similar findings were reported about Warren root collar weevils [37]. Vertical trunk silhouettes were used as visual cues for landing by bark beetles and traps incorporating appropriate visual silhouettes were more efficient [61].

Insects have evolved alternative visual strategies for guiding their behavior and locomotion in their respective environments. Unlike the need for three dimensional perception and evaluation in flying insects, the horizontal plane of the eye may be more important in crawling bed bugs because they cannot leave the surface they walk on. Like many other insects [62], the frontal visual field of bed bug eyes may be sensing and compensating the image motion of the object as they approach. The distance to an object may be evaluated by tracking the image expansion of the object, enabling them to turn away, or orient toward that object similar to that described in other insects [63]. The 30 cm tall vertical objects did not produce any additional stimulus than that from 15 cm tall objects in our study. This may be due to the fact that bed bugs have a certain threshold for the image expansion and when the image expansion exceeds that threshold, additional height did not enhance their stimulus.

We showed that bed bugs can differentiate colors and detect vertical objects at very low background light intensity (0.05 lx), which is similar to 0.01–0.1 lx light intensity during the night in the bedroom of a typical one bedroom (63 m^2^) apartment using a 13 W fluorescent lamp. Therefore, bed bug vision appears to play an important role in their host-searching and harborage-searching behavior.

It is known that bed bugs have a preference for coarse surfaces (such as paper, fabric, and wood) over smooth surfaces (such as metal and plastic) [44]. We revealed that even subtle differences in texture affect behavior of bed bugs. Bed bugs did not prefer very coarse fabric (felt) or hard texture (painted tape and textured painted plastic). The rough but hard surfaces may be hard to grasp by bed bugs claws. Very coarse fabric may tangle the bed bug legs and render their movement more difficult. A recent study showed microfabricated surface that mimic bean leaves with hooked hairs (trichomes) could entangle, but not physically entrap bed bugs [64]. The texture of the paper surgical tape provided enough traction for bed bugs to engage the surface with their tarsal claws. Adding alcohol-based black dye did not alter the texture of the paper surgical tape, whereas, adding black paint made the paper tape noticeably smoother and thus less desirable for bed bug locomotion. These findings indicate that the substrate texture can significantly affect bed bug orientation behavior, thus texture can be manipulated to improve the efficacy of bed bug monitors. Different colors, vertical objects, and textures evaluated in this study closely simulate to those encountered in the natural habitat of bed bugs. From a practical stand point, these findings are constructive for developing more effective trapping devices and designing better treatment strategies. Our studies suggest that bed bugs use vision and mechanoreception in host-seeking and harborage-searching behavior. Future research should investigate how many spectral types of photoreceptors are involved in the vision of bed bugs. Data on photoreceptor spectral sensitivities would be combined with behavioral experiments to study the cellular level mechanisms underlying vision in bed bugs.
